# Changes in Stoichiometry, Cellular RNA, and Alkaline Phosphatase Activity of *Chlamydomonas* in Response to Temperature and Nutrients

**DOI:** 10.3389/fmicb.2017.00018

**Published:** 2017-01-23

**Authors:** Dag O. Hessen, Ola T. Hafslund, Tom Andersen, Catharina Broch, Nita K. Shala, Marcin W. Wojewodzic

**Affiliations:** Aquatic Ecology and Toxicology, Department of Biosciences, University of OsloOslo, Norway

**Keywords:** alkaline phosphatase, cell size, growth, phosphorus, phytoplankton, RNA, stoichiometry, temperature

## Abstract

Phytoplankton may respond both to elevated temperatures and reduced nutrients by changing their cellular stoichiometry and cell sizes. Since increased temperatures often cause increased thermal stratification and reduced vertical flux of nutrients into the mixed zone, it is difficult to disentangle these drivers in nature. In this study, we used a factorial design with high and low levels of phosphorus (P) and high and low temperature to assess responses in cellular stoichiometry, levels of RNA, and alkaline phosphatase activity (APA) in the chlorophyte *Chlamydomonas reinhardtii.* Growth rate, C:P, C:N, N:P, RNA, and APA all responded primarily to P treatment, but except for N:P and APA, also temperature contributed significantly. For RNA, the contribution from temperature was particularly strong with higher cellular levels of RNA at low temperatures, suggesting a compensatory allocation to ribosomes to maintain protein synthesis and growth. These experiments suggest that although P-limitation is the major determinant of growth rate and cellular stoichiometry, there are pronounced effects of temperature also via interaction with P. At the ecosystem level, nutrients and temperature will thus interact, but temperatures would likely exert a stronger impact on these phytoplankton traits indirectly via its force on stratification regimes and vertical nutrient fluxes.

## Introduction

Elemental composition and temperature are key factors that affect growth and stoichiometry in algae. The ambient concentrations and ratios of key elements, such as carbon (C), nitrogen (N), and phosphorus (P) will have major impacts of phytoplankton elemental ratios and thus growth (Sterner and Elser, 2002; Klausmeier et al., 2008). Nutrient uptake and demands in autotrophs do also depend on ambient temperatures. The direct responses of temperature related to growth rate and stoichiometry are primarily governed by kinetic responses, i.e., enzyme activity, cell division, and nutrient uptake that may occur at higher rates with elevated temperature. However, also macromolecular make-up, rate of protein synthesis, and storage of elements may respond to temperature, which in this way also indirectly affect growth and cellular stoichiometry (Woods et al., 2003; Klausmeier et al., 2004; Toseland et al., 2013).

In higher plants and multicellular algae, there has been observed a general decline in specific N and P contents when moving from cold, high latitudes, toward the warmer, equatorial regions (Reich and Oleksyn, 2004; Borer et al., 2013). The decrease in P content with elevated temperatures is higher than that of N, however, causing an increase in the overall N:P ratio with increased temperature (or decreased latitude). Several studies have revealed a similar positive correlation between the overall N:P ratio of marine phytoplankton and global temperature (Martiny et al., 2013; Toseland et al., 2013; Yvon-Durocher et al., 2015), but there are few comparative lake studies, despite the fact that a strong increase in lake temperatures has been recorded worldwide (O’Reilly et al., 2015). A higher N:P with elevated temperature is likely associated with the increased enzyme efficiency at higher temperatures that cause a lower cellular density of P-rich ribosomes because fewer ribosomes are then needed to maintain a certain level of protein synthesis (Toseland et al., 2013; Thrane et al., 2016, 2017). If true, levels of RNA would also be reduced with elevated temperature, but again this response could be confounded by ambient nutrient concentrations.

Decreased phytoplankton cell size is another proposed response to warming (Atkinson et al., 2003; Daufresne et al., 2009; Sheridan and Bickford, 2011; Forster et al., 2012). The causality for smaller cell size, notably at the intraspecific level, remains obscure however. Experimental studies on phytoplankton indicate contributions both from ambient nutrient levels and temperature *per se* to cell size (Peter and Sommer, 2013, 2015), but it is difficult to disentangle the drivers based on *in situ* studies because warming also will affect thermal stratification, mixing depth, and thus vertical nutrient fluxes in aquatic ecosystems (Galbraith and Martiny, 2015). Reduced concentrations of ambient nutrients in response of reduced mixing would promote smaller cells owing to their higher surface-to-volume ratios and thus higher nutrient affinities (Raven, 1998; Marañón et al., 2012; Marañón, 2015). Hence in a “global change” context, both temperature and nutrient fluxes will change, with expected effects on the stoichiometry, growth and size of phytoplankton, yet likely with several confounding interactions (Sommer et al., 2016).

With this study, we aim to disentangle the effects of temperature and nutrients on phytoplankton growth and stoichiometry under controlled experimental conditions. To assess the responses in stoichiometry and growth, and the related responses [RNA, alkaline phosphatase activity (APA), and cell size] we conducted a factorial experiment with the chlorophyte *Chlamydomonas reinhardtii*, under high and low temperatures and high and low concentrations of phosphorus.

## Materials and Methods

For the experiments, we used the unicellular chlorophyte *C. reinhardtii* (strain CC-1690 wild type mt+) obtained from the Chlamydomonas Resource Centre (University of Minnesota). The species, and notably this strain, is widely used for experimental studies. While this species clearly may not be representative for all phytoplankton responses, it is commonly found across a variety of freshwater habitats and widely used also in ecologically relevant experiments.

The experiment was designed as a cross factorial setup with two P treatments (5 μmol P L^-1^ or 25 μmol P L^-1^), hereafter low P (LP) and high P (HP), and two temperature treatments (13 or 19°C), designated low temperature (LT) and high temperature (HT), respectively. While the concentrations of P only differ by a factor of 5, the use of chemostats and turbidostats produced P-limited and P-sufficient cultures by design (see details below), and hence the actual P-concentrations were not critical in this context. A wider temperature gradient would likely provide stronger temperature responses, but the applied temperature represent a “realistic” span in epilimnetic summer temperatures of temperate lakes. Each treatment had three replicates. The experiments were run as semicontinuous cultures in 40 ml tissue bottles (Nunclon Delta filtercap, Thermo Scientific). We used a modified version of Guillard and Lorenzen’s (1972) WC medium with filtered water from a high-alkalinity lake as a base to minimize the risk of CO_2_-deficiency. Excess N was ensured by keeping N:P well above Redfield ratio (Redfield, 1958). A concentration of 1000 μmol NO_3_ was used in both the high and LP treatments yielding molar N:P-ratios of 40:1 and 200:1, respectively. The lake water was initially filtered on Whatman GF/F and then sterile filtered (0.2 μm pore width) prior to additions of macronutrients, trace elements, and vitamins according to the WC medium recipe.

The algae were cultivated in two climate-controlled rooms of LT and HT (13 and 19°C, respectively) with a 12:12 h light-dark cycle and a light insity of approximately 85 μE m^-2^ s^-1^ of PAR (both cool and warm white light). For the LP treatment, a semicontinuous culture with a fixed dilution of 50% 3 days per week was applied. In this chemostat-type of dilution the algae are kept in a stationary growth phase below the carrying capacity. For the HP treatment we used a turbidostat-type of dilution where the culture was diluted to a fixed cell number (50,000 cells ml^-1^) 3 days per week. The turbidostat design is beneficial by a maintaining a fixed density of algae in a non-limited condition with regard to nutrients, light and CO_2_, thus avoiding the pitfalls of high-nutrient chemostats.

For all dilutions, the cultures were transferred to new bottles to avoid or minimize “bottle effects” like wall growth. Analysis of cell number (for growth estimates) and cell size were done at each day of dilution (3 days a week), samples for elemental ratios (C:N:P) were taken 14 days after inoculation, while samples for RNA and APA were taken 21 days after inoculation. However, because the APA results for two of the replicates in the HT × LP treatment had to be discarded due to a mistake made in the experimental procedure, we included a second sampling and analysis both for APA and RNA.

The cell number and size was measured by an electronic cell counter (CASY TT, Schärfe, Germany). A regular 12:12 h light:dark cycle was applied to synchronize cell division, and samples for estimations of cell densities and sizes were taken at the same time point each harvesting day. The specific growth rate was determined as the ln of relative change in cell abundance between two points in time (see Supplement for formula), and averaged for the duration of the experiment. As measures of cell size, we recorded both the mean and the peak (mode) of the size distribution of the algal samples.

For analysis of particulate C and N content, algae were collected on a GF/C filter (Whatman, Sigma-Aldrich), and analyzed using an element analyzer (Thermo Finnegan EA 1112 series flash, Thermo Fisher scientific). P-content was estimated by digesting the samples in a solution of potassium peroxydisulfate (K_2_S_2_O_8_) before colorimetric analyses using an autoanalyzer (Bran Luebbe, Norderstedt Germany). In this case, the samples were soaked in 10 ml of a 1% solution of potassium peroxydisulfate for 30 min at 120°C.

Cellular contents of RNA were included in the study both as a proxy of growth rate and to judge the effect of P-limitation. In addition, the total RNA serves as an indicator of the amount of ribosomal RNA in the cell (Flynn et al., 2010). For the RNA analyses, we applied a modified version of the RiboGreen fluorescence protocol (Turner BioSystems) (cf. Gorokhova and Kyle, 2002). Depending on cell density, we sampled 1–4 ml from each culture. The sampled volume was filtered on a nitrocellulose membrane (0.65 μm DAWP, Millipore) and the filter stored in nuclease tubes before snap-frozen in liquid N. Prior to analysis, 120 μl of the extraction buffer was added (1% sarcosyl, Sigma) and, while still frozen, the samples were homogenized by ice-cold sonification (Branson Sonifier) for 2 min. The samples were again put on ice and diluted with TE buffer in a 1:5 ratio (10 mM Tris-HCL, 1 mM EDTA, pH 7.5). For each sample, we extracted 2 × 75 μl into two separate slots of a 96-well plate (655076 Greiner Bio-one, USA). The duplicates were inserted pairwise in the columns following the first, which was reserved for standard. In the first set of duplicate samples, we added a total of 20 μl RNase-free water (Gibco BRL1071), and in the other set of duplicates 20 μl of 0.1% RNase A (A7973, Promega). Immediately after the RNase mixture was added, the well plate was incubated at 37°C on a shaking table with the output 400RPM to ensure homogenous dispatch of the RNase and digestion of RNA. After the incubation, 75 μl of 100 × diluted RiboGreen dye (R-11490, Molecular Probes, USA) was added to each well by the use of an automatic eight-channel pipette. The RNA content was then analyzed by the use of a fluorescence microplate reader (Synergy MX, BioTek Instruments, USA) with an excitation wavelength of 480 nm and an emission wavelength of 525 nm.

Alkaline phosphatase activity was included in the study as an independent biomarker for P-limitation (Thingstad and Mantoura, 2005; Litchman and Nguyen, 2008; Wang and Liang, 2014). This enzyme is used to split the ester bound in phosphomonoesters, thus removing phosphate groups from macromolecules scaled with the degree of P-deficiency (Hoppe, 2003). APA was analyzed by the CDP-star chemo-luminescence method according to the protocol of Wojewodzic et al. (2011). Samples for APA were collected as in the RNA analysis and stored at -80°C prior to analysis. For analysis, 0.3 ml of Triton X-100 solution (T8787, Sigma) was added to each sample (while kept on ice) and then sonified corresponding to the RNA procedure described above. The standards for the calibration curve were then prepared by using a concentration gradient AP type VII-S from bovine intestinal mucosa (P5521, Sigma). After the preparation of the standards, 20 μl of both the standards and the samples was transferred to a pyrophosphate-free 96-well plate (Nunc, 236105) kept on ice. Next 20 μl of the 0.4 mM CDP-star solution was dispensed with and automatic eight-channel pipette to all the wells. The APA contents of the cells were then analyzed using a fluorescence microplate reader (Synergy Mx, BioTek Instruments, USA). All statistical analysis and plotting were performed in the R programming environment v3.2 (R Development Core Team, 2016). The full script with additional data is given in the Supplementary Material.

## Results

**FIGURE 1:**
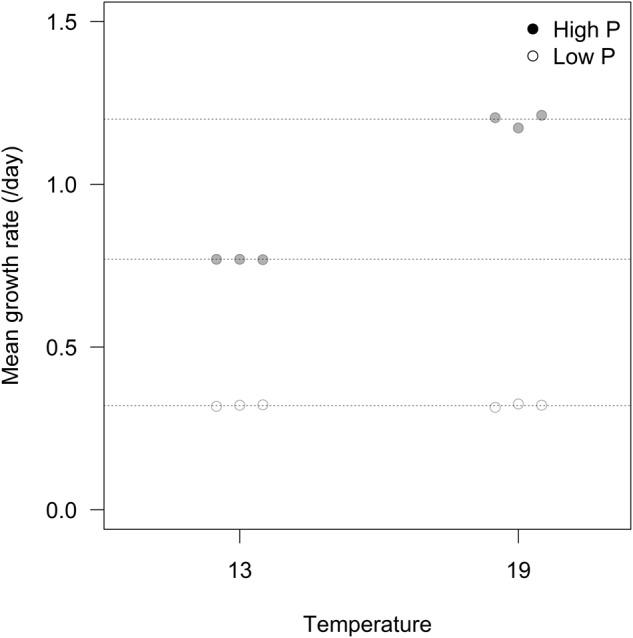
**Mean growth rates for the three replicates in each of the experimental treatments**.

We found an overall strong effect of P-treatment on growth rates of algae, but with temperature and the interaction between P-treatment and temperature as important explanatory factors contributing to differences in growth (**Figure 1**; **Table 1**). At HP × HT, growth rates stabilized at ca. 1.2 d^-1^, dropped to 0.77 d^-1^ at HP × LT, and further to 0.32 d^-1^ in the LP treatments. The growth rate responses remained stable during the 3 weeks course of the experiments (see Supplementary Figure S1).

**Table 1:** Fraction of total variance explained by temperature and P limitation treatments, and their interaction.

Variable	Growth rate (per day)	RNA (mg/ww)	Alkaline phosphatase activity (APA) (pU/ww)
P treatment	0.83	0.47	0.94
Temperature	0.08	0.21	–
P treatment × Temp.	0.08	–	–
*R*^2^	0.99	0.68	0.94

*w:w = weight fraction of wet weight (mg:mg), with wet weight estimated from total biovolume under the assumption of density = 1 g/cm^3^. 1 pU = the amount of AP enzyme required to hydrolyse 1 pmol of 4-nitrophenyl phosphate per minute at pH 9.8 at 37°C. The analysis of RNA and APA includes the results from two sampling times, and the two response variables are in both cases corrected for cell size. See supplementary for the full ANOVA table.*

**FIGURE 2:**
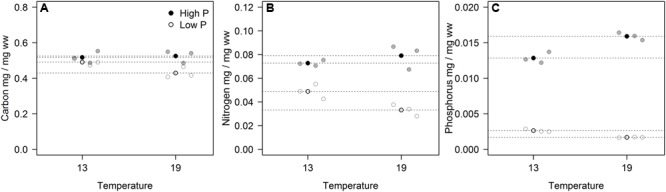
**Volume corrected contents of carbon (A)**, nitrogen **(B)**, and phosphorus **(C)** expressed as fraction of wet weight for each of the three replicates (gray circles) in the four experimental treatments. Points in black and dashed horizontal lines denote the mean in each group.

The cell-specific content of C, N, and P all responded to P treatments, yet in different fashions (**Figure 2**; **Table 2**). The response in cell-specific C was modest, yet with somewhat lower C-content at LP × HT (**Figure 2A**). The same pattern was seen for cellular N, although in this case response was stronger, notably at HT, where cell-specific N was twice as high in the HP-treatment (**Figure 2B**), and for cellular P the differences were even stronger (**Figure 2C**). For N and P, there was also a positive interaction with temperature, where HP × HT yielded the highest cell-specific content of nutrients. This effect was most prominent for P. Since the cell sizes differed somewhat between treatments, and notably with reduced cell size at HT × HP (cf. Supplementary Figure S5), the estimates of cell specific elemental contents were corrected for cell size (using the mean of the cell size distribution). The correction was also applied for the other cell-specific responses (RNA and APA). The cell-specific results (not corrected for size) are given in the supplementary in the plot to the left in Supplementary Figures S6–S8 and S13 and S14. The size correction considerably changed the patterns for the C, N, and P contents, whereas RNA and APA basically displayed the same patterns as the non-corrected.

**Table 2:** The proportion of variance explained (significantly) by the experimental variables.

Variable	C content (mg/ww)	N content (mg/ww)	P content (mg/ww)	C:N (molar)	C:P (molar)	N:P (molar)
P treatment	0.47	0.82	0.96	0.79	0.93	0.97
Temperature	–	0.01	0.01	0.03	0.03	–
P treatment × Temp.	–	0.08	0.03	0.08	0.04	–
*R*^2^	0.47	0.91	0.99	0.90	0.99	0.97

*See supplementary for the full ANOVA table. The nutrient contents are corrected for cell size (ww, wet weight), and the nutrients ratios are in molar units.*

**FIGURE 3:**
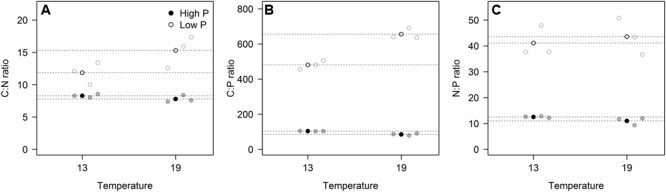
**The elemental ratios for carbon:nitrogen (A)**, carbon:phosphorus **(B)**, and nitrogen:phosphorus **(C)** for each of the three replicates (gray circles) in the four experimental treatments. Points in black and dashed horizontal lines denote the mean in each group. All ratios in molar units.

These responses to the T × P treatments yielded strong responses in elemental ratios, and unsurprisingly with the largest deviation in C:P, primarily in response to P-treatment, and for C:N and C:P also with modest contribution from temperature (**Figures 3A–C**; **Table 2**). The responses in N:P was consistent across temperature treatments with a fourfold decrease in N:P in the HP treatments. Also C:N responded strongly with lower ratios in the HP treatments, likely as a response to higher N-demands for protein synthesis with high access to P. In this case temperature also contributed, notably in the LP treatment with elevated C:N.

**FIGURE 4:**
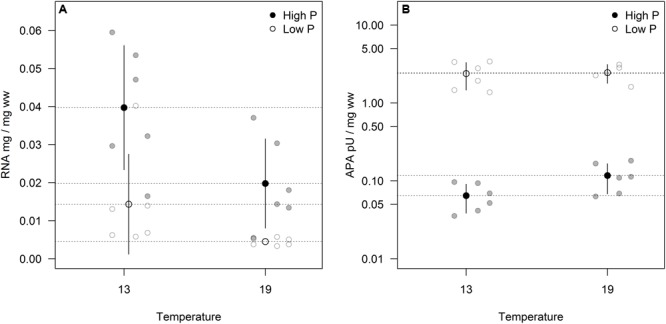
**Volume corrected RNA contents (A)** and alkaline phosphatase activity (APA)/enzyme content **(B)** expressed as fraction of wet weight for two sampling times. Points in black and dashed horizontal lines denote the mean in each group (vertical lines = standard deviation).

Cellular RNA responded strongly both to temperature and phosphorus with higher levels at HP and LT (**Figure 4A**; **Table 1**). While RNA corresponded well with cellular P (**Figure 2C**), it deviated from the growth rate responses (cf. **Figure 1**). Note that despite higher growth rates at HT (19°C), the RNA-concentration was higher at LT (13°C). APA ranged almost two orders of magnitude in response to P-treatment, with almost negligible contributions from temperature (**Figure 4B**; **Table 1**).

**FIGURE 5:**
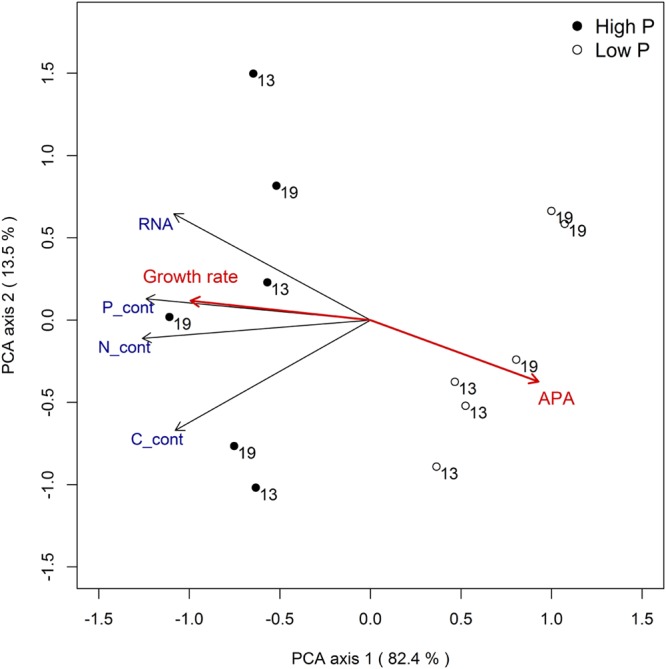
**Principal component analysis for responses related to RNA and elemental content (size corrected) with growth rate and APA as passive variables**.

The combined response for all variables to temperature and phosphorus treatments can be summarized by a principal component analysis (**Figure 5**). The analysis clearly supports that P treatment was the experimental factor that explained most of the variation in RNA and nutrient contents (the separation of points along PCA axis 1, which captures 82% of the variance, primarily reflects P treatment). Growth rate and APA activity is included in the plot as passive variables (i.e., they did not influence the ordination) and clearly convey a strong positive relationship between P availability and growth rate, and a negative one between P availability and APA.

## Discussion

The overall conclusion from these experiments is that P-limitation is the major determinant of growth rate as well as stoichiometry and indicators thereof (RNA and APA), but that temperature also exerted a significant impact on most parameters. The cultures were maintained as chemostats (although semicontinuous) and turbidostats for the LP and HP treatments, respectively, to ensure chronically P-deficient and P-saturated cells. The APA analysis clearly showed that these premises were fulfilled with a striking deviation between HP and LP cultures under both temperatures. While APA only marginally responded to temperature, suggesting that this enzymatic response is not sensitive to a temperature span from 13 to 19°C, cellular RNA was much elevated at LT under both HP and LP, indicating a compensatory mechanism to maintain the rate of protein synthesis and thus growth at reduced temperature. Still growth rates were consistently lower at LT for the HP treatment (while not for LP).

The responses in RNA were also reflected in the cell quotas of P, and hence also the cellular stoichiometry. It is however noteworthy that the response in C:P was not only related to cellular quotas of P, but also of C (**Figure 2A**). The fact that also C:N were impacted by elevated P likely reflects its effect on growth and protein synthesis. Cellular processes, such as transcription and translation require a coupling of N and P, where P is needed for mRNA synthesis while N is required for protein synthesis.

The causal relationship between the close correlations typically found between growth rate, P-content and RNA in small heterotrophs, such as bacteria, crustacean zooplankton, and other invertebrates (Elser et al., 2000), is not straightforward, i.e., high growth rate could promote high RNA-content, but also be a consequence of other factors promoting elevated growth rates (in both cases this presupposes that sufficient P is available for making RNA). Also the availability of N would modify the relationship between RNA and growth rate, since the rate of protein synthesis may be constrained by the access to N (amino acids).

The contrasting temperature responses we found between cell-specific P-content relative to that of RNA is striking, however, and with the proviso that we here only tested one species, and that cryophilic species might respond differently, this support the “RNA-efficiency” hypothesis (cf. Woods et al., 2003; Cotner et al., 2006). This means higher demands of RNA at low temperature to maintain the levels of protein synthesis. This may further result in decreased N:P-ratios at lower temperatures, or thus *vice versa* as a response of warming (Martiny et al., 2013; Toseland et al., 2013; Yvon-Durocher et al., 2015), which is supported by the observed trend of decreased N:P in foliage or macroalgae when moving pole-wards (Reich and Oleksyn, 2004; Borer et al., 2013). For pelagic autotrophs, however, the stoichiometric responses may be confounded by the aforementioned change in mixing regimes along temperature gradients, and our study on *Chlamydomonas* suggest no impact at all of temperature on N:P. The chlorophyte *Chlamydomonas* may, however, not necessarily be representative of responses in other taxa. Different species and taxa of phytoplankton may have different strategies and responses with regard to N and P acquisition together with the N:P ratio of nutrient availability (Klausmeier et al., 2004; Thrane et al., 2016). In a more detailed assay with the same strain of *Chlamydomonas* grown in microwells along a wide gradient of temperatures and ambient N:P in the media, the optimum N:P ratio shifted from 27 to 37 (atomic ratio) over a temperature gradient from 11 to 18°C (Thrane et al., 2017). Yet, as pointed out in their paper, there are differences in measured optimal demand ratios for maximum growth and the ratio of total cellular pools of N:P in cells. Very strong deviations in N:P should basically not be expected, however, due to the mutual demands for N and P under transcription and translation as well as other cellular processes. I.e., also APA may ultimately be influenced by N-availability (Marklein and Houlton, 2012), and while photosynthetic capacity may be limited by the high N-demands of the photosynthetic machinery, so may P-limitation constrain the production of Rubisco (Reich et al., 2009). Corresponding temperature responses in stoichiometry and RNA has been observed in heterotrophic bacteria (Cotner et al., 2006).

Among the parameters tested in this study, RNA gave the strongest temperature response with elevated cellular concentrations at LT, but also growth rate and C:P showed a relatively strong response to temperature. Since growth rate was positively related to temperature, especially at HP (**Figure 1**), this does indeed suggest a stronger allocation of P to RNA under low temperature. The fact that it did not result in a corresponding decrease in N:P suggest that N-uptake and protein synthesis kept pace with P-uptake and RNA synthesis. RNA did not correspond well with growth rate across temperatures, and to which extent the growth rate hypothesis (GRH) holds for autotrophs is still a matter of controversy (Ågren, 2004; Matzek and Vitousek, 2009; Flynn et al., 2010). It is, however, important to note that the GRH was explicitly formulated to hold within temperatures (Elser et al., 2000), hence within temperature (either 13 or 19°C) growth rate clearly match RNA content (and P).

Whether or not temperature *per se* affect cell size, or indirectly via lower ambient nutrient concentrations, is an issue of major concern in the context of global warming. In our experiments, smaller cells were found under HP × HT, but this response likely reflects the higher rate of cell division in this treatment. Still this implies that systems with higher temperatures could give smaller cells, but only when sufficient nutrients are available to promote strong growth.

Although temperature poses a direct impact on phytoplankton traits, notably on growth rate and cellular RNA, judged from these experiments, temperature would be expected to exert the strongest impact on phytoplankton indirectly via changes in stratification regimes and vertical nutrient fluxes.

## Author Contributions

DH had the idea of the experiment, and planned the design together with TA. OH was the main responsible for conducting the experiment with help from CB, NS, and MW, and was also instrumental in the analysis of the data together with input from the other authors. CB run the final scripts, stats, and figures in discussion with TA and DH. DH wrote up the manuscript with input from all co-authors.

## Conflict of Interest Statement

The authors declare that the research was conducted in the absence of any commercial or financial relationships that could be construed as a potential conflict of interest.
