# Supplementary material for: Changes in Stoichiometry, Cellular RNA, and Alkaline Phosphatase Activity of Chlamydomonas in Response to Temperature and Nutrients
Source: Front Microbiol. 2017 Jan 23;8:18. doi: 10.3389/fmicb.2017.00018 (PMC5253361; doi:10.3389/fmicb.2017.00018)
Supplement: Supplementary file 1 [file Presentation_1.zip › supplementary_Hessen_2016_frontiers.html]

### Supplementary material

## **Changes in Stoichiometry, Cellular RNA and Alkaline Phosphatase Activity of *Chlamydomonas* in Response to Temperature and Nutrients**

#### Dag O. Hessen, Ola T. Hafslund, Tom Andersen, Catharina Broch, Nita K. Shala and Marcin Wojewodzic

This supplementary material provides the raw data, the transformations, calculations and the essential code that underlie the results presented in the figures in the journal article (the code for plotting is, however, hidden). The document is rendered in R Markdown.

#### **Contents**

GROWTH

NUTRIENTS

Contents

Ratios (molar)  
- Carbon:Nitrogen  
- Nitrogen:Phosphorus  
- Carbon:Phosphorus

RNA CONTENTS

ALKALINE PHOSPHATASE

PCA

(this list of contents works as hyperlinks - click to go to section)

### GROWTH

Estimations of cell number (growth) and cell size were done every second or third day in the experimental period.

### Growth rates

Formula used to calculate growth rates:

\(Growth \: rate=\frac{ln(\displaystyle \frac{N\_{t\_2}}{N\_{t\_1}})}{t\_2 - t\_1}\)

where *N* denotes the population density (concentration of algae) at time *t*.

#### **Figure S1.** Intrinsic growth rates estimated at different time points in the experimental period at 13 degrees (left) and 19 degrees (right) for the three replicates in the high phosphorus (solid points) and low phosphorus (open points) treatment.

#### Calculated means for each replicate (object *growth2*)

```
##    mean_rates Temperature Diet
## 1   0.3178168          13  low
## 2   0.3213270          13  low
## 3   0.3224174          13  low
## 4   0.7693270          13 high
## 5   0.7695102          13 high
## 6   0.7681948          13 high
## 7   0.3147791          19  low
## 8   0.3251146          19  low
## 9   0.3214822          19  low
## 10  1.2046796          19 high
## 11  1.1730282          19 high
## 12  1.2120286          19 high
```

#### **Figure S2.** Mean growth rates for the three replicates in each of the experimental treatments. This figure is equal to Fig 1. in the journal article.

#### Analyse differences between treatments

```
mod8 <- lm(mean_rates ~ factor(Temperature) * Diet, data=growth2)
anova(mod8)
```

```
## Analysis of Variance Table
## 
## Response: mean_rates
##                          Df  Sum Sq Mean Sq F value    Pr(>F)    
## factor(Temperature)       1 0.13707 0.13707  1183.6 5.571e-10 ***
## Diet                      1 1.31594 1.31594 11362.6 6.702e-14 ***
## factor(Temperature):Diet  1 0.13715 0.13715  1184.2 5.558e-10 ***
## Residuals                 8 0.00093 0.00012                      
## ---
## Signif. codes:  0 '***' 0.001 '**' 0.01 '*' 0.05 '.' 0.1 ' ' 1
```

P treatment explains most of the variation (0.83), temperature explains 0.09, and the interaction 0.09.

```
summary(mod8)
```

```
## 
## Call:
## lm(formula = mean_rates ~ factor(Temperature) * Diet, data = growth2)
## 
## Residuals:
##       Min        1Q    Median        3Q       Max 
## -0.023551 -0.001288  0.000653  0.002587  0.015450 
## 
## Coefficients:
##                                Estimate Std. Error t value Pr(>|t|)    
## (Intercept)                    0.769011   0.006213  123.77 2.03e-14 ***
## factor(Temperature)19          0.427568   0.008787   48.66 3.52e-11 ***
## Dietlow                       -0.448490   0.008787  -51.04 2.40e-11 ***
## factor(Temperature)19:Dietlow -0.427630   0.012427  -34.41 5.56e-10 ***
## ---
## Signif. codes:  0 '***' 0.001 '**' 0.01 '*' 0.05 '.' 0.1 ' ' 1
## 
## Residual standard error: 0.01076 on 8 degrees of freedom
## Multiple R-squared:  0.9994, Adjusted R-squared:  0.9992 
## F-statistic:  4577 on 3 and 8 DF,  p-value: 2.829e-13
```

At the low nutrient treatment, the mean growth rate is equal at 13 and 19 degrees (0.32/day). At the high P treatment, the mean growth rate is 0.77/day at 13 degrees and 1.2/day at 19.

### Cell sizes

As measures of cell size, we recorded both the mean and the peak (mode) of the algal sample’s size distribution.

#### **Figure S3.** The mean diameter of all the cells sampled from the population at different time points in the experimental period for the three replicates in the high phosphorus (solid circles) and low phosphorus treatment (open circles) at 13 (left) and 19 degrees (right).

#### **Figure S4.** The peak diameter (mode) of all the cells sampled from the population at different time points in the experimental period for the three replicates in the high phosphorus (solid circles) and low phosphorus treatment (open circles) at 13 (left) and 19 degrees (right).

#### Calculated means in **mean** diameter for each replicate (object *cellsize\_mean*)

```
##    mean_meansize Temperature Diet
## 1          5.919          13  low
## 2          5.996          13  low
## 3          5.996          13  low
## 4          6.378          13 high
## 5          6.406          13 high
## 6          6.448          13 high
## 7          6.523          19  low
## 8          6.540          19  low
## 9          6.549          19  low
## 10         5.146          19 high
## 11         5.162          19 high
## 12         5.156          19 high
```

#### Calculated means in **peak** diameter (mode) for each replicate (object *cellsize\_peak*)

```
##    mean_peaksize Temperature Diet
## 1       5.342000          13  low
## 2       5.336000          13  low
## 3       5.370000          13  low
## 4       5.482000          13 high
## 5       5.481000          13 high
## 6       5.621000          13 high
## 7       5.747000          19  low
## 8       5.761000          19  low
## 9       5.791111          19  low
## 10      4.855000          19 high
## 11      4.895000          19 high
## 12      4.995000          19 high
```

#### **Figure S5.** Calculated means of the mean (left) and peak (right) diameter of all the cells sampled from the population for the three replicates in each experimental treatment.

#### Analyse differences in **mean** size between treatments

```
mod.cellmean <- lm(mean_meansize ~ factor(Temperature) * Diet, data=cellsize_mean)
koef.cellmean <- coef(mod.cellmean)

summary(mod.cellmean)
```

```
## 
## Call:
## lm(formula = mean_meansize ~ factor(Temperature) * Diet, data = cellsize_mean)
## 
## Residuals:
##      Min       1Q   Median       3Q      Max 
## -0.05133 -0.01008  0.00200  0.01517  0.03733 
## 
## Coefficients:
##                               Estimate Std. Error t value Pr(>|t|)    
## (Intercept)                    6.41067    0.01697  377.68  < 2e-16 ***
## factor(Temperature)19         -1.25600    0.02400  -52.32 1.97e-11 ***
## Dietlow                       -0.44033    0.02400  -18.34 8.03e-08 ***
## factor(Temperature)19:Dietlow  1.82300    0.03395   53.70 1.60e-11 ***
## ---
## Signif. codes:  0 '***' 0.001 '**' 0.01 '*' 0.05 '.' 0.1 ' ' 1
## 
## Residual standard error: 0.0294 on 8 degrees of freedom
## Multiple R-squared:  0.998,  Adjusted R-squared:  0.9973 
## F-statistic:  1355 on 3 and 8 DF,  p-value: 3.656e-11
```

At 13 degrees and low P, the mean cell size is 6 µm and at high P 6.4 µm. At 19 degrees and low P, the mean cell size is 6.5 µm and at high P 5.2 µm.

#### Analyse differences in **peak** size between treatments

```
mod.cellpeak <- lm(mean_peaksize ~ factor(Temperature) * Diet, data=cellsize_peak)
koef.cellpeak <- coef(mod.cellpeak)

summary(mod.cellpeak)
```

```
## 
## Call:
## lm(formula = mean_peaksize ~ factor(Temperature) * Diet, data = cellsize_peak)
## 
## Residuals:
##      Min       1Q   Median       3Q      Max 
## -0.06000 -0.02650 -0.01033  0.02168  0.09300 
## 
## Coefficients:
##                               Estimate Std. Error t value Pr(>|t|)    
## (Intercept)                    5.52800    0.03231 171.112 1.52e-15 ***
## factor(Temperature)19         -0.61300    0.04569 -13.417 9.12e-07 ***
## Dietlow                       -0.17867    0.04569  -3.911  0.00448 ** 
## factor(Temperature)19:Dietlow  1.03004    0.06461  15.942 2.40e-07 ***
## ---
## Signif. codes:  0 '***' 0.001 '**' 0.01 '*' 0.05 '.' 0.1 ' ' 1
## 
## Residual standard error: 0.05596 on 8 degrees of freedom
## Multiple R-squared:  0.9789, Adjusted R-squared:  0.971 
## F-statistic: 123.9 on 3 and 8 DF,  p-value: 4.807e-07
```

At 13 degrees and low P, the peak cell size is 5.3 µm and at high P 5.5 µm. At 19 degrees and low P, the mean cell size is 5.8 µm and at high P 4.9 µm.

### NUTRIENTS

```
CNP <- read.table("CNP contents.txt", header=T)
kable(CNP)
```

| Temperature\_C | Diet | Replicate | cells\_on\_filter | ug\_P\_filter | ug\_N\_filter | ug\_C\_filter |
| --- | --- | --- | --- | --- | --- | --- |
| 19 | high | 1 | 1931430 | 2.273 | 12 | 76 |
| 19 | high | 2 | 2067480 | 2.365 | 10 | 72 |
| 19 | high | 3 | 2010480 | 2.215 | 12 | 78 |
| 19 | low | 1 | 1995650 | 0.479 | 11 | 119 |
| 19 | low | 2 | 1810650 | 0.459 | 9 | 123 |
| 19 | low | 3 | 1953900 | 0.482 | 8 | 119 |
| 13 | high | 1 | 902940 | 1.575 | 9 | 64 |
| 13 | high | 2 | 924240 | 1.554 | 9 | 62 |
| 13 | high | 3 | 865540 | 1.637 | 9 | 66 |
| 13 | low | 1 | 2378520 | 0.763 | 13 | 135 |
| 13 | low | 2 | 2446920 | 0.692 | 15 | 129 |
| 13 | low | 3 | 2527245 | 0.703 | 12 | 138 |

Calculate from **µg**/filter to **mg**/cell

```
CNP$mg_P_cell <- (CNP$ug_P_filter/10^3)/CNP$cells_on_filter
CNP$mg_N_cell <- (CNP$ug_N_filter/10^3)/CNP$cells_on_filter
CNP$mg_C_cell <- (CNP$ug_C_filter/10^3)/CNP$cells_on_filter

# and from µg/filter to mg/filter
CNP$mg_P_filter <- CNP$ug_P_filter/10^3
CNP$mg_N_filter <- CNP$ug_N_filter/10^3
CNP$mg_C_filter <- CNP$ug_C_filter/10^3
```

Calculate from **µg**/filter to **mol**/cell

```
# Molar masses
mmP <- 30.97 # Phosphorus g/mol
mmN <- 14.01 # Nitrogen g/mol
mmC <- 12.01 # Carbon g/mol

CNP$mol_P_filter <- ((CNP$ug_P_filter/10^6)/mmP)  
CNP$mol_N_filter <- ((CNP$ug_N_filter/10^6)/mmN) 
CNP$mol_C_filter <- ((CNP$ug_C_filter/10^6)/mmC) 
# Since molar mass is given in units of g/mol, we divide by 10^6 to convert from µg to g. 

CNP$mol_P_cell <- CNP$mol_P_filter/CNP$cells_on_filter
CNP$mol_N_cell <- CNP$mol_N_filter/CNP$cells_on_filter
CNP$mol_C_cell <- CNP$mol_C_filter/CNP$cells_on_filter
```

### Contents

### Phosphorus content

#### Normalise contents to fraction of wet weight

For all cell volume normalisations (estimations of the elemental content per fraction of wet weight), we use the formula for a volume of a sphere:

\(V = \frac{4}{3} \pi r^3\)

together with the model for the measured mean diameter in each treatment (object *cellsize* below) and the assumption that the algal density = 1 g/ cm3.

```
# extract coefficient values from model of mean cell size
cellsize <- c(rep(koef.cellmean[1]+koef.cellmean[2],3),
              rep(koef.cellmean[1]+koef.cellmean[2]+koef.cellmean[3]+koef.cellmean[4],3), 
              rep(koef.cellmean[1],3), 
              rep(koef.cellmean[1]+koef.cellmean[3],3))
              # 19 high P, 19 low P, 13 high P, 13 low P

CNP$mg_P_normalizcell <- CNP$mg_P_filter/((((4/3)*pi*(cellsize/2)^3)*10^-9)*CNP$cells_on_filter)
```

#### **Figure S6.** Volume corrected phosphorus contents expressed as fraction of wet weight (left; equal to Fig. 2c in the article) and cell specific phosphorus contents that is not corrected for cell volume (right) for each of the three replicates (grey circles) in the four experimental treatments. Points in black and dashed horizontal lines denote the mean in each group.

#### Analyse differences between treatments

```
mod1 <- lm(mg_P_normalizcell ~ Diet * factor(Temperature), data=CNP)

anova(mod1)
```

```
## Analysis of Variance Table
## 
## Response: mg_P_normalizcell
##                          Df     Sum Sq    Mean Sq  F value    Pr(>F)    
## Diet                      1 0.00044768 0.00044768 1921.830 8.088e-11 ***
## factor(Temperature)       1 0.00000334 0.00000334   14.321  0.005355 ** 
## Diet:factor(Temperature)  1 0.00001207 0.00001207   51.803 9.268e-05 ***
## Residuals                 8 0.00000186 0.00000023                       
## ---
## Signif. codes:  0 '***' 0.001 '**' 0.01 '*' 0.05 '.' 0.1 ' ' 1
```

P treatment explains most of the variation in P content (0.96). The other effects are also statistically significant but with small effect size (fraction explained: temperature = 0.01 and the interaction effect = 0.03).

### Nitrogen content

#### Normalise contents to fraction of wet weight

(see explanation for normalisation of P content)

```
CNP$mg_N_normalizcell <- CNP$mg_N_filter/((((4/3)*pi*(cellsize/2)^3)*10^-9)*CNP$cells_on_filter)
```

#### **Figure S7.** Volume corrected nitrogen contents expressed as fraction of wet weight (left; equal to Fig. 2b in the article) and cell specific nitrogen contents that is not corrected for cell volume (right) for each of the three replicates (grey circles) in the four experimental treatments. Points in black and dashed horizontal lines denote the mean in each group.

#### Analyse differences between treatments

```
mod2 <- lm(mg_N_normalizcell ~ Diet * factor(Temperature), data=CNP)
anova(mod2)
```

```
## Analysis of Variance Table
## 
## Response: mg_N_normalizcell
##                          Df    Sum Sq   Mean Sq F value    Pr(>F)    
## Diet                      1 0.0036475 0.0036475 84.2754 1.601e-05 ***
## factor(Temperature)       1 0.0000651 0.0000651  1.5033   0.25502    
## Diet:factor(Temperature)  1 0.0003643 0.0003643  8.4169   0.01986 *  
## Residuals                 8 0.0003462 0.0000433                      
## ---
## Signif. codes:  0 '***' 0.001 '**' 0.01 '*' 0.05 '.' 0.1 ' ' 1
```

P treatment explains most of the variation in N content (0.82), temperature explains 0.01 and the interaction 0.08.

### Carbon content

#### Normalise contents to fraction of wet weight

(see explanation for normalisation of P content)

```
CNP$mg_C_normalizcell <- CNP$mg_C_filter/((((4/3)*pi*(cellsize/2)^3)*10^-9)*CNP$cells_on_filter)
```

#### **Figure S8.** Volume corrected carbon contents expressed as fraction of wet weight (left; equal to Fig. 2a in the article) and cell specific carbon contents that is not corrected for cell volume (right) for each of the three replicates (grey circles) in the four experimental treatments. Points in black and dashed horizontal lines denote the mean in each group.

#### Analyse differences between treatments

```
mod3 <- lm(mg_C_normalizcell ~ Diet, data=CNP)
anova(mod3)
```

```
## Analysis of Variance Table
## 
## Response: mg_C_normalizcell
##           Df   Sum Sq   Mean Sq F value  Pr(>F)  
## Diet       1 0.011244 0.0112437  8.7397 0.01438 *
## Residuals 10 0.012865 0.0012865                  
## ---
## Signif. codes:  0 '***' 0.001 '**' 0.01 '*' 0.05 '.' 0.1 ' ' 1
```

Only P treatment explains variation in carbon content (R2 = 0.47).

### Ratios (molar)

### Carbon:Nitrogen

#### **Figure S9.** The carbon : nitrogen ratio for each of the three replicates (grey circles) in the four experimental treatments. Points in black and dashed horizontal lines denote the mean in each group. This figure is equal to Fig. 3a in the journal article.

The average C:N ratio is about 8 for the high P treatments, and for the low P treatments about 12 at 13 degrees and 15 at 19 degrees. The variance is heteroscedastic, we therefore transform data (log) before analysis.

#### Analyse differences between treatments

```
mod4 <- lm(log(mol_C_cell/mol_N_cell)~ factor(Temperature_C) * Diet, data=CNP)
anova(mod4)
```

```
## Analysis of Variance Table
## 
## Response: log(mol_C_cell/mol_N_cell)
##                            Df  Sum Sq Mean Sq F value    Pr(>F)    
## factor(Temperature_C)       1 0.02699 0.02699  1.9808   0.19696    
## Diet                        1 0.77755 0.77755 57.0548 6.587e-05 ***
## factor(Temperature_C):Diet  1 0.07580 0.07580  5.5620   0.04607 *  
## Residuals                   8 0.10902 0.01363                      
## ---
## Signif. codes:  0 '***' 0.001 '**' 0.01 '*' 0.05 '.' 0.1 ' ' 1
```

P treatment explains most of the variation in the C:N ratio (0.79), temperature explains 0.03, and the interaction between them explains 0.08.

### Nitrogen:Phosphorus

#### **Figure S10.** The nitrogen : phosphorus ratio for each of the three replicates (grey circles) in the four experimental treatments. Points in black and dashed horizontal lines denote the mean in each group. This figure is equal to Fig. 3c in the journal article.

The average N:P ratios in the high P treatments are about 13 (13 degrees) and 11 (19 degrees), and for the low P treatments about 41 (13 degrees) and 44 (19 degrees). Variance is not equal between the high and low P treatments, we therefore transform data (log) before analysis.

#### Analyse differences between treatments

```
mod5 <- lm(log(mol_N_cell/mol_P_cell) ~ Diet, data=CNP)
anova(mod5)
```

```
## Analysis of Variance Table
## 
## Response: log(mol_N_cell/mol_P_cell)
##           Df Sum Sq Mean Sq F value    Pr(>F)    
## Diet       1 4.9013  4.9013     302 8.438e-09 ***
## Residuals 10 0.1623  0.0162                      
## ---
## Signif. codes:  0 '***' 0.001 '**' 0.01 '*' 0.05 '.' 0.1 ' ' 1
```

P treatment explains most of the variation in N:P ratios (R2 0.97).

### Carbon:Phosphorus

#### **Figure S11.** The carbon : phosphorus ratio for each of the three replicates (grey circles) in the four experimental treatments. Points in black and dashed horizontal lines denote the mean in each group.This figure is equal to Fig. 3b in the journal article.

The average C:P ratios in the high P treatments are 104 (13 degrees) and 85 (19 degrees). In the low P treatments the average ratios are 481 (13 degrees) and 656 (19 degrees). Differences in variance are not too bad. We perform analysis without transformation.

#### Analyse differences between treatments

```
mod6 <- lm(mol_C_cell/mol_P_cell ~ factor(Temperature_C) * Diet, data=CNP)
anova(mod6)
```

```
## Analysis of Variance Table
## 
## Response: mol_C_cell/mol_P_cell
##                            Df Sum Sq Mean Sq  F value    Pr(>F)    
## factor(Temperature_C)       1  18333   18333   46.359 0.0001366 ***
## Diet                        1 674169  674169 1704.802 1.304e-10 ***
## factor(Temperature_C):Diet  1  28153   28153   71.192 2.971e-05 ***
## Residuals                   8   3164     395                       
## ---
## Signif. codes:  0 '***' 0.001 '**' 0.01 '*' 0.05 '.' 0.1 ' ' 1
```

P treatment explains most of the variation in the C:P ratio (0.93), temperature explains 0.03, and the interaction between them explains 0.04.

### RNA CONTENTS

```
RNA <- read.table("RNA content.txt", header=T, dec=",")
kable(RNA)
```

| sample\_time | Temperature\_C | Diet | Replicate | Cell\_conc\_ml | Sample\_size\_ml | ug\_RNA |
| --- | --- | --- | --- | --- | --- | --- |
| first | 19 | high | 1 | 359983 | 1.4 | 0.1977835 |
| first | 19 | high | 2 | 380417 | 1.4 | 0.5477655 |
| first | 19 | high | 3 | 372617 | 1.4 | 0.5010689 |
| first | 19 | low | 1 | 333975 | 1.4 | 0.3600097 |
| first | 19 | low | 2 | 351725 | 1.4 | 0.4118768 |
| first | 19 | low | 3 | 350050 | 1.4 | 0.3614639 |
| first | 13 | high | 1 | 106413 | 4.2 | 3.6676912 |
| first | 13 | high | 2 | 122850 | 4.2 | 3.8048293 |
| first | 13 | high | 3 | 117675 | 4.2 | 2.1958758 |
| first | 13 | low | 1 | 253400 | 2.0 | 0.7379087 |
| first | 13 | low | 2 | 268400 | 2.0 | 2.4024171 |
| first | 13 | low | 3 | 260175 | 2.0 | 0.8073119 |
| second | 19 | high | 1 | 747945 | 0.7 | 1.3903991 |
| second | 19 | high | 2 | 779739 | 0.7 | 1.1870276 |
| second | 19 | high | 3 | 799689 | 0.7 | 0.7235609 |
| second | 19 | low | 1 | 576706 | 0.9 | 0.2864101 |
| second | 19 | low | 2 | 590706 | 0.9 | 0.2586991 |
| second | 19 | low | 3 | 562039 | 0.9 | 0.2785734 |
| second | 13 | high | 1 | 284664 | 1.8 | 2.0947740 |
| second | 13 | high | 2 | 292089 | 1.8 | 3.4121005 |
| second | 13 | high | 3 | 289889 | 1.8 | 1.1821894 |
| second | 13 | low | 1 | 487639 | 1.1 | 0.3699469 |
| second | 13 | low | 2 | 479139 | 1.1 | 0.3402969 |
| second | 13 | low | 3 | 491406 | 1.1 | 0.4102610 |

Calculate from **µg**/sample to **g**/cell

```
# Cells per sample
RNA$cells_sample <- RNA$Cell_conc_ml*RNA$Sample_size_ml

# RNA per cell
RNA$g_RNA_cell <- (RNA$ug_RNA*10^-6)/RNA$cells_sample
```

#### Subset groups and normalise contents to fraction of wet weight

(see explanation for normalisation of P content)

```
RNA_all <- RNA
RNA_all$g_RNA_normalizcell <- (RNA_all$ug_RNA*10^-3)/((((4/3)*pi*(cellsize/2)^3)*10^-9)*RNA_all$cells_sample)

RNA1 <- subset(RNA, sample_time == "first" )
RNA1$g_RNA_normalizcell <- (RNA1$ug_RNA*10^-3)/((((4/3)*pi*(cellsize/2)^3)*10^-9)*RNA1$cells_sample)

RNA2 <- subset(RNA, sample_time == "second" )
RNA2$g_RNA_normalizcell <- (RNA2$ug_RNA*10^-3)/((((4/3)*pi*(cellsize/2)^3)*10^-9)*RNA2$cells_sample)
```

#### **Figure S12.** Volume corrected RNA contents expressed as fraction of wet weight for the first (left) and second (right) sampling time for the three replicates (grey circles) in the four experimental treatments. Points in black and dashed horizontal lines denote the mean in each group (vertical lines = standard deviation).

#### **Figure S13.** Volume corrected RNA contents expressed as fraction of wet weight (left; equal to Fig. 4a in the article) and cell specific RNA contents that is not corrected for cell volume (right) for both sampling times. Points in black and dashed horizontal lines denote the mean in each group (vertical lines = standard deviation).

#### Analyse differences between treatments

```
modRNA <- lm(log(g_RNA_normalizcell) ~ factor(Temperature_C) + Diet, data=RNA_all) 
anova(modRNA)
```

```
## Analysis of Variance Table
## 
## Response: log(g_RNA_normalizcell)
##                       Df Sum Sq Mean Sq F value    Pr(>F)    
## factor(Temperature_C)  1 4.3521  4.3521  14.137  0.001152 ** 
## Diet                   1 9.5970  9.5970  31.174 1.533e-05 ***
## Residuals             21 6.4648  0.3078                      
## ---
## Signif. codes:  0 '***' 0.001 '**' 0.01 '*' 0.05 '.' 0.1 ' ' 1
```

P treatment explains 0.47 and temperature 0.21 of the variation in RNA content.

### ALKALINE PHOSPHATASE

```
APA <- read.table("Alkaline phosphatase.txt", header=T, dec=",")
kable(APA)
```

| sample\_time | Temperature\_C | Diet | Replicate | Cell\_conc\_ml | Sample\_size\_ml | uU\_APA |
| --- | --- | --- | --- | --- | --- | --- |
| first | 19 | high | 1 | 337263 | 1.4 | 0.0010067 |
| first | 19 | high | 2 | 355930 | 1.4 | 0.0012219 |
| first | 19 | high | 3 | 361697 | 1.4 | 0.0020725 |
| first | 19 | low | 1 | 355330 | 1.4 | 0.0090911 |
| first | 19 | low | 2 | 343655 | 1.4 | 0.1051741 |
| first | 19 | low | 3 | 362030 | 1.4 | 0.0726159 |
| first | 13 | high | 1 | 131980 | 4.0 | 0.0037016 |
| first | 13 | high | 2 | 129605 | 4.0 | 0.0034539 |
| first | 13 | high | 3 | 121580 | 4.0 | 0.0022500 |
| first | 13 | low | 1 | 249555 | 2.0 | 0.0405488 |
| first | 13 | low | 2 | 260530 | 2.0 | 0.0583123 |
| first | 13 | low | 3 | 260030 | 2.0 | 0.0413885 |
| second | 19 | high | 1 | 747945 | 0.7 | 0.0032758 |
| second | 19 | high | 2 | 779739 | 0.7 | 0.0023347 |
| second | 19 | high | 3 | 799689 | 0.7 | 0.0040794 |
| second | 19 | low | 1 | 576706 | 0.9 | 0.0890119 |
| second | 19 | low | 2 | 590706 | 0.9 | 0.1171007 |
| second | 19 | low | 3 | 562039 | 0.9 | 0.0602858 |
| second | 13 | high | 1 | 284664 | 1.8 | 0.0012807 |
| second | 13 | high | 2 | 292089 | 1.8 | 0.0015781 |
| second | 13 | high | 3 | 289889 | 1.8 | 0.0019457 |
| second | 13 | low | 1 | 487639 | 1.1 | 0.1073631 |
| second | 13 | low | 2 | 479139 | 1.1 | 0.0863287 |
| second | 13 | low | 3 | 491406 | 1.1 | 0.1109203 |

Calculate from **µU**/sample to **U**/cell

```
# Cells per sample
APA$cells_sample <- APA$Cell_conc_ml*APA$Sample_size_ml

# APA per cell
APA$U_APA_cell <- (APA$uU_APA*10^-6)/APA$cells_sample

# We did a mistake in the experimental procedure, so we regard the results for the following two datapoints as false
APA$U_APA[4] <- NA
APA$U_APA[6] <- NA
```

#### Subset groups and normalise contents to activity/enzyme content per wet weight

```
APA_all <- APA
APA_all$U_APA_normalizcell <- (APA_all$U_APA)/((((4/3)*pi*(cellsize/2)^3)*10^-9)*APA_all$cells_sample)

APA1 <- subset(APA, sample_time == "first" )
APA1$U_APA_normalizcell <- (APA1$U_APA)/((((4/3)*pi*(cellsize/2)^3)*10^-9)*APA1$cells_sample)

APA2 <- subset(APA, sample_time == "second" )
APA2$U_APA_normalizcell <- (APA2$U_APA)/((((4/3)*pi*(cellsize/2)^3)*10^-9)*APA2$cells_sample)
```

#### **Figure S14.** Volume corrected APA activity/enzyme contents expressed as fraction of wet weight for the first (left) and second (right) sampling time for the three replicates (grey circles) in the four experimental treatments. Points in black and dashed horizontal lines denote the mean in each group. In the first sampling, we lost the results for two of the replicates in the high temperature x low phosphorus treatment.

#### **Figure S15.** Volume corrected APA activity/enzyme content expressed as fraction of wet weight (left; equal to Fig. 4b in the article) and cell specific APA that is not corrected for cell volume (right) for both sampling times. Points in black and dashed horizontal lines denote the mean in each group (vertical lines = standard deviation). In the first sampling, we lost the results for two of the replicates in the high temperature x low phosphorus treatment.

```
modAPA <- lm(log(U_APA_normalizcell) ~ Diet, data=APA_all)
anova(modAPA)
```

```
## Analysis of Variance Table
## 
## Response: log(U_APA_normalizcell)
##           Df Sum Sq Mean Sq F value    Pr(>F)    
## Diet       1 61.057  61.057  307.55 1.306e-13 ***
## Residuals 20  3.971   0.199                      
## ---
## Signif. codes:  0 '***' 0.001 '**' 0.01 '*' 0.05 '.' 0.1 ' ' 1
```

P treatment explains 0.94 of the variation in APA.

## PCA

The code for the analysis is given below, while the code for the plotting is hidden. We used the results from the second sampling time for RNA and APA.

```
library(vegan)

# active variables
X <- data.frame(CNP$mg_C_normalizcell, CNP$mg_N_normalizcell, CNP$mg_P_normalizcell, RNA2$g_RNA_normalizcell)
varnames <- c("C_cont", "N_cont", "P_cont", "RNA")

pca.X <- rda(X, scale=T) # scale to unit variance

prop.explained <- summary(eigenvals(pca.X))
prop.explained <- prop.explained$importance
prop.explained
```

```
##                            PC1       PC2       PC3        PC4
## Eigenvalue            3.294554 0.5397609 0.1188614 0.04682342
## Proportion Explained  0.823640 0.1349400 0.0297200 0.01171000
## Cumulative Proportion 0.823640 0.9585800 0.9882900 1.00000000
```

```
# passive variables
Y <- data.frame(APA2$U_APA_normalizcell, growth2$mean_rates)
colnames(Y) <- c("APA", "Growth rate")

XY <- envfit(pca.X, Y, perm=999)

XY$vectors
```

```
##                  PC1      PC2     r2 Pr(>r)   
## APA          0.92759 -0.37361 0.7866  0.004 **
## Growth rate -0.99305  0.11771 0.8222  0.003 **
## ---
## Signif. codes:  0 '***' 0.001 '**' 0.01 '*' 0.05 '.' 0.1 ' ' 1
## Permutation: free
## Number of permutations: 999
```

#### **Figure S16.** PCA plot with contents of carbon, nitrogen, phosphorus and RNA (volume corrected) as active variables (influencing the ordination of the replicates for all treatments), together with APA and growth rate as passive variables. This figure is equal to Figure 5 in the journal article.
